# Brain regions that support accurate speech production after damage to Broca’s area

**DOI:** 10.1093/braincomms/fcab230

**Published:** 2021-10-01

**Authors:** Diego L Lorca-Puls, Andrea Gajardo-Vidal, PLORAS Team, Marion Oberhuber, Susan Prejawa, Thomas M H Hope, Alexander P Leff, David W Green, Cathy J Price

**Keywords:** stroke, aphasia, speech production, Broca’s area, cerebellum

## Abstract

Broca’s area in the posterior half of the left inferior frontal gyrus has traditionally been considered an important node in the speech production network. Nevertheless, recovery of speech production has been reported, to different degrees, within a few months of damage to Broca’s area. Importantly, contemporary evidence suggests that, within Broca’s area, its posterior part (i.e. pars opercularis) plays a more prominent role in speech production than its anterior part (i.e. pars triangularis). In this study, we therefore investigated the brain activation patterns that underlie accurate speech production following stroke damage to the opercular part of Broca’s area. By combining functional MRI and 13 tasks that place varying demands on speech production, brain activation was compared in (i) seven patients of interest with damage to the opercular part of Broca’s area; (ii) 55 neurologically intact controls; and (iii) 28 patient controls with left-hemisphere damage that spared Broca’s area. When producing accurate overt speech responses, the patients with damage to the left pars opercularis activated a substantial portion of the normal bilaterally distributed system. Within this system, there was a lesion-site-dependent effect in a specific part of the right cerebellar Crus I where activation was significantly higher in the patients with damage to the left pars opercularis compared to both neurologically intact and patient controls. In addition, activation in the right pars opercularis was significantly higher in the patients with damage to the left pars opercularis relative to neurologically intact controls but not patient controls (after adjusting for differences in lesion size). By further examining how right Crus I and right pars opercularis responded across a range of conditions in the neurologically intact controls, we suggest that these regions play distinct roles in domain-general cognitive control. Finally, we show that enhanced activation in the right pars opercularis cannot be explained by release from an inhibitory relationship with the left pars opercularis (i.e. dis-inhibition) because right pars opercularis activation was positively related to left pars opercularis activation in neurologically intact controls. Our findings motivate and guide future studies to investigate (i) how exactly right Crus I and right pars opercularis support accurate speech production after damage to the opercular part of Broca’s area and (ii) whether non-invasive neurostimulation to one or both of these regions boosts speech production recovery after damage to the opercular part of Broca’s area.

## Graphical Abstract

**Graphical Abstract:**
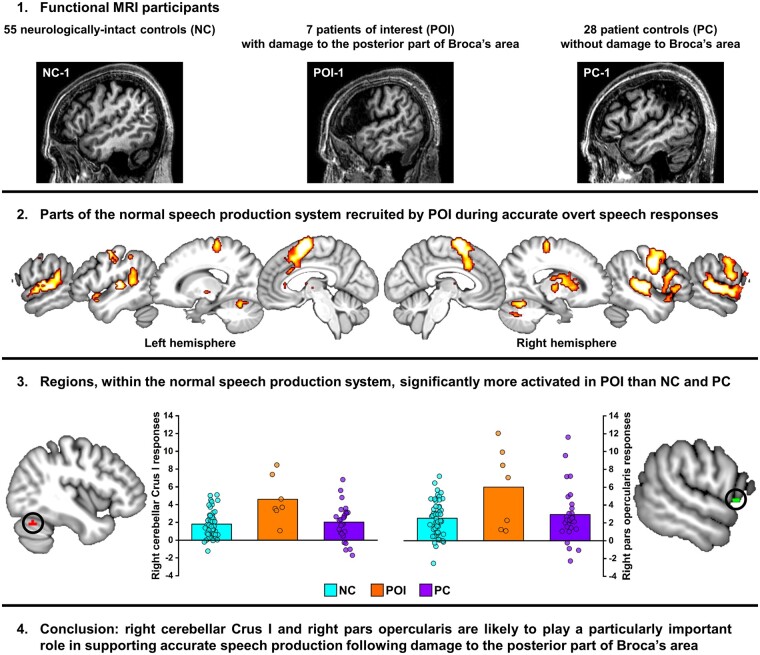

## Teaser

By combining functional neuroimaging and a wide range of tasks that place varying demands on speech production, Lorca-Puls et al. reveal that right cerebellar Crus I and right pars opercularis are likely to play a particularly important role in supporting successful speech production following damage to Broca’s area.

## Introduction

Paul Broca’s seminal work in the 1860s attributed a critical role in speech production to the posterior half of the left inferior frontal gyrus.^1–3^ In honour of Broca’s novel contribution, this part of the left frontal lobe has, ever since, been known as Broca’s area. It is commonly defined as the combination of the pars triangularis (or Brodmann area 45) and the pars opercularis (or Brodmann area 44). However, recent functional MRI (fMRI) work in neurologically intact individuals revealed that, within Broca’s area, only the pars opercularis tracks the demands on speech production.^4^ Indeed, Mugler et al.^5^ showed in patients undergoing awake craniotomy for glioma removal that activity patterns in the left pars opercularis (LpOp) encode representations of speech sounds that are both context-independent (phonemes) and context-dependent (articulatory gestures). Moreover, in a study of awake neurosurgical patients, by Long et al.,^6^ focal cooling of LpOp disrupted speech production. Critically, although damage to LpOp has been associated with speech production impairments in the acute phase post-stroke,^7^ we previously reported that LpOp damage does not contribute to persistent speech production impairments, when the effect of co-occurring white matter damage in the vicinity of the anterior arcuate fasciculus is taken into account.^8^ Together, prior evidence leads us to hypothesize that other brain regions can compensate for the effect of LpOp damage on speech production.

In the present fMRI study, we investigated which brain regions support accurate overt speech production in stroke survivors with relatively circumscribed left frontal damage involving the opercular part of Broca’s area. This is in contrast to the vast majority of previous functional neuroimaging studies in which patients with heterogeneous lesion locations were pooled together. Instead, our work draws from several emerging sources of evidence indicating that the brain activation patterns underlying normal behavioural responses after stroke depend, among other factors, on the location of the lesion.^9–12^ The research question we pose is relevant because Broca’s area continues to occupy a prominent position in highly influential dual-stream models of the speech network.^13–16^ Furthermore, characterizing how accurate overt speech production is achieved in the presence of damage to LpOp will help us to understand better (i) the functional anatomy of speech production and (ii) the behavioural consequences of damage to the opercular part of Broca’s area.

Our interest in investigating the brain activation patterns that support accurate overt speech production after LpOp damage stems from the following three observations. First, an inability to accurately produce overt speech is one of the most disabling behavioural consequences of stroke, affecting the capacity of the patient to verbally express their feelings, thoughts and needs. Second, the association between damage to Broca’s area and speech production impairments has been reported to weaken with time post-stroke,^17^^,^^18^ which suggests that speech production is recovering as a result of functional reorganization and adaptive neuroplasticity. Third, identifying the brain areas that may be able to compensate for damage to the opercular part of Broca’s area is essential for indicating potential target regions for future non-invasive neurostimulation therapeutic studies.

What specific tasks should be used to identify the brain regions that support accurate overt speech production following LpOp damage? Previous studies adopted a variety of speech production tasks to probe the function of LpOp, consistent with the fact that speech production is a multifaceted human ability that requires the interaction of multiple levels of processing, such as phonetic, phonological, lexical-semantic and morphosyntactic processing. LpOp function has been assessed, for example, with the production of single words and connected speech.^7^^,^^19^ In this study, we focus on the two tasks from our multifactorial fMRI paradigm that place highest demands on phonological/phonetic encoding during speech production: pseudoword reading and pseudoword repetition. This is motivated by prior claims that LpOp is implicated in the phonological/phonetic encoding of a speech plan.^4^^,^^5^^,^^19^ For example, Flinker et al.^19^ showed in patients undergoing surgical treatment for refractory epilepsy that activation in Broca’s area (i) peaked before the onset of speech articulation; (ii) ended at the onset of speech articulation; and (iii) increased when the stimuli were pseudowords compared to real words. Other studies using fMRI in neurologically intact individuals have also convincingly demonstrated that the overt production of meaningless unfamiliar sequences of speech sounds (i.e. pseudowords) robustly recruits LpOp.^4^^,^^20–25^ More importantly, stroke-induced LpOp damage has been reported to impair the ability to read and repeat pseudowords,^26–30^ which suggests that these tasks remain challenging for patients with LpOp damage over the course of recovery.

To the best of our knowledge, only two previous functional imaging studies, by Rosen et al.^31^ and Blank et al.,^32^ investigated overt speech production in chronic stroke patients with left frontal lobe damage involving Broca’s area. In Rosen et al.,^31^ a group of six right-handed chronic stroke patients showed increased activation within the right posterior inferior frontal gyrus (RpIFG), left supplementary motor area (SMA), left pre-SMA and right Heschl's gyrus compared to a group of six neurologically intact subjects during an overt word-stem completion task (e.g. see DRA, say ‘drama’). In Blank et al.,^32^ a group of seven right-handed chronic stroke patients showed increased activation within the right pars opercularis relative to a group of twelve neurologically intact individuals during propositional speech (prompted by open-ended autobiographical questions such as ‘what do you like to do on holiday?’). However, four important design characteristics of the studies by Rosen et al.^31^ and Blank et al.^32^ motivate further investigation. First, these prior studies did not exclude patients whose lesions extended well beyond the left frontal lobe, making it challenging to infer which damaged regions triggered the reported compensatory neural changes. Second, they employed tasks that (i) are seldom, if ever, used in the assessment of acquired speech and language disorders after stroke (i.e. word-stem completion), or (ii) elicit experimentally uncontrolled responses across patients (i.e. open-ended autobiographical questions). Third, they were not able to determine whether increased activation was the consequence of accurate or inaccurate overt speech production because the imaging data were acquired using PET, which has a low temporal resolution (averaging over a minute or two) that does not permit the dissociation of correct from incorrect responses. Fourth, they were unlikely to be able to capture the normal range of variability in brain responses given the low number of neurologically intact participants included in the control group (*n* = 6 and 12, respectively).

With the aim of building, improving and extending upon the work of Rosen et al.^31^ and Blank et al.,^32^ we: (i) selected stroke patients with left frontal lesions involving LpOp; (ii) excluded stroke patients whose lesions extended into posterior speech production regions in lateral inferior parietal or superior temporal association cortices; (iii) employed tasks (i.e. pseudoword reading and repetition) that maximize the demands on overt speech production while minimizing the demands on semantics (because pseudowords have no associated meaning); (iv) focussed on correct trials only to ensure that brain responses reflected accurate overt speech production; (v) tested a much larger group of neurologically intact subjects (*n* = 55) to better estimate the range of normal brain responses and increase statistical power; (vi) included a group of patients with lesions elsewhere in the left hemisphere (*n* = 28) to elucidate if any differences in brain activation between patients with LpOp damage and neurologically intact controls were lesion-site-dependent; (vii) characterized inter-patient variability in brain activation within the identified compensatory regions; and (viii) capitalized on the flexibility afforded by our multi-task fMRI paradigm to infer the type of function that the identified potentially compensatory regions may normally subserve.

Based on previous research findings, we hypothesized that accurate overt speech production in patients with LpOp damage would recruit a bilaterally distributed set of brain areas similar to that observed in neurologically intact individuals.^33^ We anticipated that this task-relevant neural system would likely comprise regions involved in speech production and cognitive control such as superior temporal cortex and SMA/pre-SMA, respectively.^34^^,^^35^ Within this task-relevant neural system, we hypothesized that the contralesional homologous cortex would play a particularly important role in compensating for loss of LpOp.^31^^,^^32^^,^^36–38^ Alternatively, if the effect in RpIFG reported in prior neuroimaging studies was merely driven by inaccurate overt speech responses in the patients with damage to Broca’s area, this right-hemisphere region would not be identified by our statistical analyses which focussed only on accurate overt speech responses. Our second hypothesis was that novel compensatory regions may be revealed given the methodological/experimental aspects, described above, that differentiate our study from past work.

In summary, the primary goal of the present fMRI study was to generate new insights into the neural mechanisms supporting accurate overt speech production following damage to the opercular part of Broca’s area.

## Materials and methods

### Participants

We searched the Predicting Language Outcome and Recovery After Stroke (PLORAS)^39^ database for stroke patients who had (i) participated in the fMRI component of this project and (ii) sustained unilateral damage centred on the left frontal lobe including the posterior part of Broca’s area: left pars opercularis (LpOp). To identify these patients (with damage to LpOp), a region of interest (i.e. IFG_L_6_1 and IFG_L_6_6 combined) derived from the Brainnetome Atlas was used.^40^ Subsequently, for each of the identified patients, the T_1_-weighted whole-brain image was visually inspected to confirm the presence of damage to LpOp. Finally, patients whose lesions extended into posterior speech production areas in lateral inferior parietal and superior temporal association cortices were excluded from the selected sample. Other inclusion criteria were as follows: (i) more than 50% accuracy on at least one of the two fMRI tasks of interest (described below) and no less than 25% accuracy on the other; (ii) >6 months since stroke onset; (iii) aged over 18 years at stroke onset; (iv) no history of neurological or psychiatric illness (other than stroke); (v) native speaker of English; and (vi) right-handed pre-morbidly. A binary classification of the patients’ speech production abilities into impaired or unimpaired performance was not part of the inclusion criteria because even those patients with impaired speech production abilities will sometimes be able to produce accurate overt speech responses. By focussing on these accurate overt speech responses, we should therefore be able to reveal the brain regions that support accurate overt speech production in stroke survivors with relatively circumscribed left frontal damage involving LpOp, a brain region that has repeatedly been implicated in speech production in the prior literature. In other words, the absence of impaired speech production abilities (immediately after stroke onset or at the time of testing) in the presence of damage to a speech production region makes our research even more relevant.

**Figure 1:**
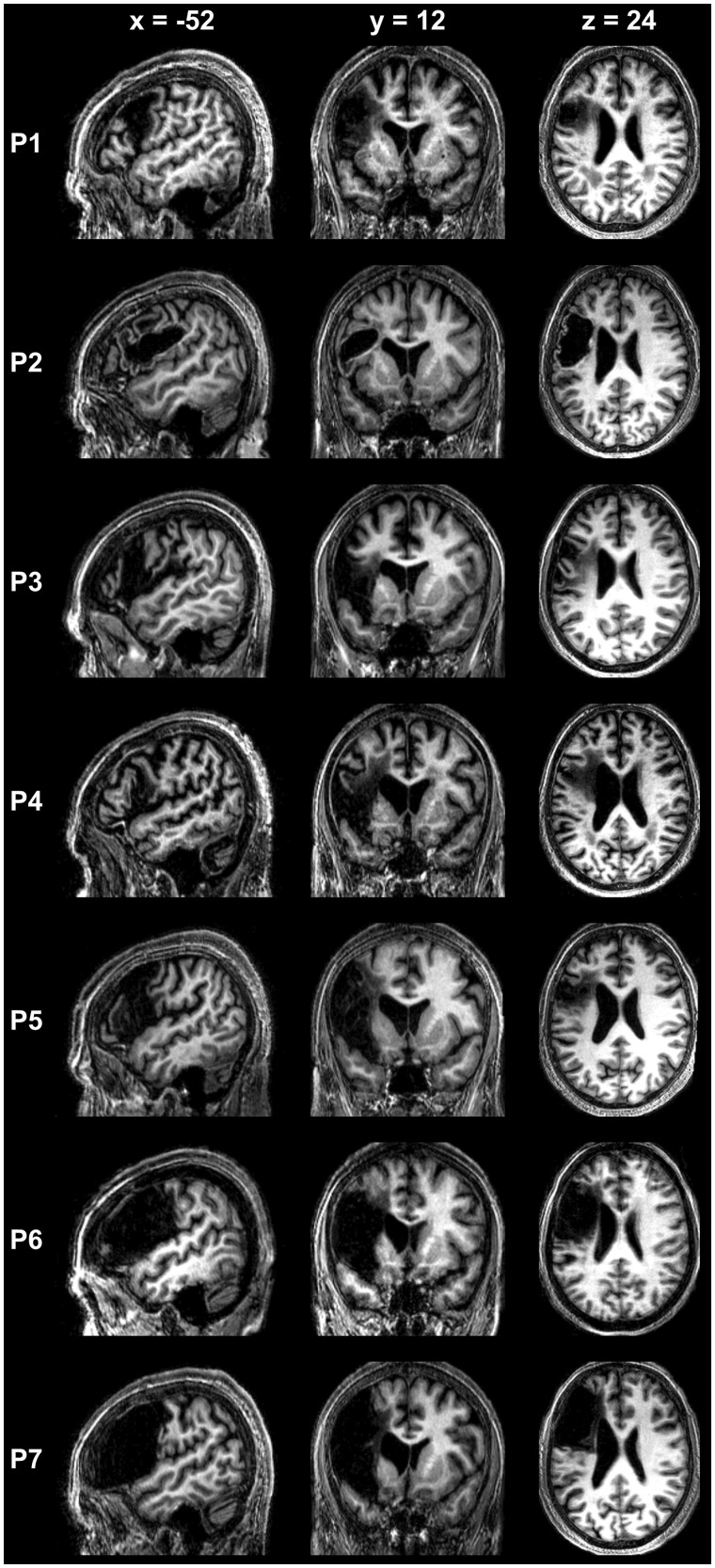
**Spatially normalized T_1_-weighted MRI scans of seven patients with LpOp damage. **Sagittal, coronal and axial views showing the location and extent of stroke damage in the seven patients of interest with LpOp damage. The scans are sorted from top to bottom by the size of the patient’s lesion (smallest lesion at the top and largest lesion at the bottom). In the PLORAS database, the following identifier was assigned to each patient: P1 = PS1414, P2 = PS0454, P3 = PS0005, P4 = PS0255, P5 = PS0426, P6 = PS0419 and P7 = PS0241.

A total of seven patients met our strict criteria and were included in the study. All were more than one-year post-stroke (i.e. in the chronic phase), and none had previously suffered another stroke. The type of stroke was ischaemic in six cases and haemorrhagic in one case (i.e. P2). Moreover, in all seven cases, damage to LpOp co-occurred with damage to other neighbouring frontal regions that previous studies have associated with speech production such as the adjacent ventrolateral premotor cortex and the underlying white matter in the vicinity of the arcuate fasciculus (see Fig. 1). Regarding the lesion status of the left pars triangularis, it was completely spared in one patient (P1), completely damaged in three patients (P5, P6 and P7), and partially damaged (i.e. only the posterior most portion) in the remaining three patients (P2, P3 and P4). Lastly, changes in the periventricular white matter consistent with leukoaraiosis were seen on the T_1_-weighted whole-brain image of two of these patients (P1 and P4).

The neurologically intact participants (*n* = 55), who were all right-handed native English speakers, served as a normative group. A third group of 28 left-hemisphere stroke patients was also included to investigate whether brain activation differences between patients with LpOp damage and neurologically intact controls were lesion-site-dependent. These ‘patient controls’ were selected based on the absence of damage to LpOp but otherwise with the same inclusion criteria (1–6 specified above) as the patients of interest with LpOp damage. All 28 patient controls were more than one-year post-stroke (i.e. in the chronic phase), and none had previously suffered another stroke. Regarding the type of stroke, 25 patient controls suffered an ischaemic insult and three patient controls suffered a haemorrhagic insult. In relation to the presence of changes in the periventricular white matter consistent with leukoaraiosis, these were seen on the T_1_-weighted whole-brain image of five of these patients. As shown in Supplementary Fig. 1, the most frequent lesion sites in the patient controls were the rostroventral supramarginal gyrus and the underlying white matter in the vicinity of the arcuate fasciculus, a region where stroke damage has frequently been associated with speech production impairments.^41^

Summary demographic, clinical and lesion information for the three participant groups is provided in Table 1. Briefly, the patients with LpOp damage were significantly older than the neurologically intact controls (*P *=* *0.005), but well matched to the patient controls (without LpOp damage) for age at scan, age at stroke and time post-stroke (all *P *>* *0.6). Therefore, any differences in brain activation between the patients with LpOp damage and controls (both neurologically intact and patient) could not be explained by any of these factors. As the patients with LpOp damage had significantly larger lesions than the patient controls (*P *=* *0.029), we considered the potential influence of lesion size on our results. For all three participant groups, in-scanner behavioural performance is summarized in Table 1 (only tasks of interest) and Supplementary Table 1 (all tasks). The scores obtained by the patients of interest (with LpOp damage) and patient controls (without LpOp damage) on a set of speech and language tasks from the Comprehensive Aphasia Test (CAT)^42^ can be found in Supplementary Table 2.

**Table 1:** Demographical, clinical and behavioural details by group

		Group		
		NC	POI	PC
		(*n* = 55)	(*n* = 7)	(*n* = 28)
Age at scan (years)	Mean(±SD)	43.4(±17.6)	63.3(±13.4)	61.3(±10.1)
	Range	20.0–75.5	43.6–83.6	40.2–75.7
Age at stroke (years)	Mean(±SD)	–	55.1(±14.5)	53.4(±12.6)
	Range	–	29.7–72.3	24.6–74.1
Time post-stroke (months)	Mean(±SD)	–	98.8(±38.9)	95.7(±71.5)
	Range	–	57.3–165.8	15.3–275.6
Lesion size (cm^3^)	Mean(±SD)	–	79.9(±45.4)	39.1(±41.8)
	Range	–	40.0–156.9	0.0^a^–159.8
Sex	Females	33	3	10
	Males	22	4	18
Handedness		All RH	All RH	All RH
PsRd accuracy (%)	Mean(±SD)	98.1(±3.3)	82.1(±18.7)	87.7(±15.0)
	Range	85.0–100.0	42.5–100.0	50.0–100.0
PsRp accuracy (%)	Mean(±SD)	97.7(±4.5)	83.9(±11.9)	84.6(±14.4)
	Range	82.5–100.0	67.5–97.5	45.0–100.0
PsRd RTs (seconds)	Mean(±SD)	0.85(±0.16)	1.22(±0.20)	1.07(±0.18)
	Range	0.51–1.22	1.01–1.56	0.84–1.51
PsRp RTs (seconds)	Mean(±SD)	1.25(±0.17)	1.40(±0.22)	1.36(±0.20)
	Range	0.88–1.63	1.17–1.77	1.11–1.98

NC, neurologically intact controls; POI, patients of interest (with LpOp damage); PC, patient controls (without LpOp damage); RH, right-handed; PsRd, pseudoword reading; PsRp, pseudoword repetition; RTs, response times.

a Two patient controls with small left subcortical lacunes (one putaminal and one thalamic) that our automated lesion identification procedure could not segment.

The study was approved by the London Queen Square Research Ethics Committee. All participants gave written informed consent prior to enrolment and were compensated £10 per hour for their time. The experimental procedures were in accordance with the Declaration of Helsinki.

### fMRI experimental design

All participants performed the same fMRI paradigm comprising 13 different tasks, in two consecutive experiments (Experiment 1 and Experiment 2). The flexibility afforded by our multi-task fMRI paradigm therefore allows a wide range of research questions to be investigated by focussing on a specific combination of tasks. In this study, the paradigm was used to investigate the function of regions that showed decreased/increased activation during accurate pseudoword production following LpOp damage relative to neurologically intact and patient controls.

Our tasks of interest were pseudoword reading and repetition. Data from the remaining 11 tasks in Experiment 1 and 2 are reported to aid the interpretation of the results obtained from the pseudoword tasks of interest. The first experiment with five tasks (Experiment 1) has already been fully described, including stimuli characteristics, in Sanjuán et al.^43^ who investigated how two neighbouring subregions in the lateral anterior temporal lobe contribute to semantic matching and object naming. The second experiment with eight tasks (Experiment 2) has been fully described in Oberhuber et al.^44^ who investigated functional subdivisions within the left supramarginal gyrus during single-word production. Our interest in the pseudoword reading and repetition tasks from Experiment 2 is that both these tasks were expected to robustly engage the opercular part of Broca’s area. The pseudoword reading task involved reading aloud unfamiliar written words (e.g. ‘dees’). The pseudoword repetition task involved repeating aloud unfamiliar spoken words (e.g. ‘fint’).

Together with pseudoword reading and pseudoword repetition, Experiment 2 comprised a 2×2×2 factorial design that tested speech production in response to stimuli that varied in sensory modality (auditory versus visual), semantic content (words and objects > pseudowords and baselines) and phonological content (words and pseudowords > objects and baselines). This allowed us to tease apart activation related to perceptual, semantic and phonological processing in terms of behavioural and neural response. The word production tasks involved reading or repeating aloud familiar written or spoken object names, respectively. The object naming tasks entailed either naming objects from pictures (visual modality) or from the sounds they make (auditory modality). The baseline tasks entailed either naming the colour of meaningless patterns or naming the gender of the voice humming a meaningless unfamiliar rhythm with no speech or semantics. Accuracy scores and response times (RTs) from the eight tasks in Experiment 2 were analysed with repeated measures ANOVAs (see Supplementary Table 3) to segregate the effects of phonology, semantics and modality.

The five tasks in Experiment 1 presented pictures of two objects or pairs of spoken object names. The four tasks that presented pictures of two objects involved: (i) naming the two objects; (ii) producing a short sentence to describe how the two objects were interacting (e.g. ‘The cat is drinking from a jug’); (iii) naming the verb describing the action between the two objects (‘drinking’); or (iv) silently making semantic matching decisions (related versus unrelated objects) on the pictures. The fifth task presented pairs of spoken object names and entailed silently making semantic matching decisions (related versus unrelated objects) on the heard object names. This combination of tasks enables us to identify activation related to noun versus verb naming, sentence production and semantic associations while controlling for the perceptual content of the stimuli.

All participants completed the tasks in the same order and the stimuli used for each task were kept constant across participants. This ensures that any group differences cannot be explained by differences in experimental variables (tasks and stimuli).

### Stimulus creation and presentation

Pseudowords (e.g. ‘vop’) were created using a nonword generator.^45^ A total of 40 pseudowords were selected after piloting; 20 had one syllable and 20 had two syllables. Both pseudoword tasks (reading and repeating) were assigned an equal number of pseudowords (20 each), with half being one-syllable in length and half two-syllable. The stimuli for the pseudoword repetition task were recorded by a native English speaker with a southern British accent approximating Received Pronunciation. The mean duration of the spoken pseudowords was 0.66 s with a standard deviation of 0.08 (range = 0.54–0.78). Pseudoword stimuli were matched to the word stimuli for bigram frequency, number of orthographic neighbours and spoken word length.

Prior to scanning, each participant was trained on all tasks using a separate set of stimuli until they felt comfortable with the task. Once inside the fMRI scanner, participants were asked to produce overt speech while minimizing the amount of head motion as much as possible. They were also instructed to fixate their eyes on a cross at the centre of the screen. This was monitored with eye-tracking.

Each task comprised a separate scan run with four blocks of stimuli. In Experiment 2 (which included the pseudoword reading and repetition tasks), each block presented 10 stimuli at a rate of one every 2.5 s. In Experiment 1, pairs of items were presented at half the rate (one every 5 s) resulting in 10 stimuli per block in all tasks. Every stimulus block, in all 13 tasks, was followed by 16-s resting periods, during which the participant fixated on a cross centred on the screen. These resting-with-fixation periods allowed activation to return to baseline between blocks, therefore ensuring maximum sensitivity to all effects of interest.

Immediately before the start of each stimulus block, a written instruction was displayed (e.g. ‘Read unfamiliar words’) lasting for the length of one inter-scan interval (i.e. 3.08 s), which reminded the subject of the task. Within the pseudoword reading and pseudoword repetition tasks, novel (not used in any previous trials) one-syllable and two-syllable pseudowords were presented in the first and third blocks, respectively. The second and fourth blocks presented identical stimuli as the preceding block but in a different order.

Visual stimuli were presented via an LCD projector, and an adjustable head-coil mirror, onto a screen that was clearly visible to the subject (1024×768 resolution). Text for the reading tasks was displayed in lower case Helvetica with a visual angle ranging from 1.47 to 4.41 degrees. Auditory stimuli were presented via MRI compatible headphones (MR Confon, Magdeburg, Germany), which filtered ambient in-scanner noise. Volume levels were adjusted for each subject prior to scanning. Spoken responses were recorded via a noise-cancelling MRI microphone (FOMRI III^TM^ Optoacoustics, Or-Yehuda, Israel), and transcribed manually for offline analysis.

### In-scanner behavioural data processing

Spoken responses were transcribed online and scored offline. Each response was categorized as either ‘correct’ (i.e. when the response matched the target) or ‘incorrect’ (i.e. when the response did not match the target, was delayed or self-corrected). For spoken pseudoword responses, deviations of up to one phoneme were scored as ‘correct’, because an item analysis indicated that this amount of variability in pronunciation was frequently seen in neurologically intact participants.

RTs for spoken responses were obtained from the audio files, using an adaptive moving window filter. The optimal window length (i.e. the width which maximally smoothed the audio stream) was based on a portion of the respective audio file collected during rest. After smoothing the whole time series, we defined the onset of speech as a rise in the absolute amplitude of the smoothed audio stream of at least three standard deviations from the mean.

The median RT for each task per subject was calculated based on correct responses only and submitted to behavioural data analyses (Supplementary Table 3), which were conducted in IBM SPSS Statistics for Windows, Version 26.0 (IBM Corp., Armonk, NY, USA). For four neurologically intact controls and two patients with LpOp damage, it was not possible to retrieve the RTs for specific tasks due to technical issues (see Supplementary Fig. 2 for details). Missing RTs were replaced with the mean for that task (correct trials only) from the remaining subjects in the corresponding group. In addition, one patient with LpOp damage (i.e. P4) did not complete the sentence production (from Experiment 1) and gender naming (from Experiment 2) tasks and the same approach was used to replace their missing data points during the RT analysis. Missing data imputation never occurred for more than a single participant (control or patient) in any given task per group.

### fMRI data acquisition

Functional and structural data were collected using the same acquisition sequences on two 3 T Trio scanners (Siemens Healthcare, Erlangen, Germany) with a 12-channel head coil. To minimize movement during scanning, a careful head fixation procedure with foam padding was adopted when positioning each participant’s head in the 12-channel head coil. Total scanning time was approximately 1 h and 30 min per subject, including set up and the acquisition of a structural scan.

Functional images were acquired using a gradient-echo EPI sequence: TR/TE = 3080 ms/30 ms, Flip angle = 90°, matrix size = 64×64, FOV = 192×192, slice thickness = 2 mm, inter-slice gap = 1 mm, voxel size = 3×3×3 mm^3^. The TR was chosen to maximize whole-brain coverage (44 slices) and allowed us to asynchronise the slice acquisition with stimulus onset to allow for distributed sampling of stimulus onset across slices in each task.^46^ Each functional run consisted of 66 whole-brain volumes per time series, including 5 ‘dummy scans’ to allow for magnetization to reach equilibrium.

Structural images were acquired after the EPIs, using a 3D modified driven equilibrium Fourier transform sequence^47^: TR/TE/TI = 7.92 ms/2.48 ms/910 ms, Flip angle = 16, 176 slices, voxel size = 1×1×1 mm^3^.

### fMRI data pre-processing

We performed fMRI data pre-processing and statistical analysis in SPM12 (UCL Wellcome Centre for Human Neuroimaging, London, UK), running in MATLAB (The MathWorks, Inc., Natick, MA, USA). Functional volumes were spatially realigned to the first EPI volume and unwarped. We used the unwarping procedure in preference to including the realignment parameters as covariates of no interest in the first level analysis because unwarping accounts for non-linear distortions by modelling the interaction between head movement and any inhomogeneity in the T_2_* signal. The structural T_1_-weighted image was co-registered to the mean EPI image generated during the realignment step and then spatially normalized to MNI space using the new unified normalization-segmentation routine in SPM12. To spatially register all realigned EPI scans to MNI space, we applied the deformation field parameters that were obtained during the normalization of the structural T_1_-weighted image. The original resolution of the structural and functional images was maintained during normalization (voxel size 1×1×1 mm^3^ for structural and 3×3×3 mm^3^ for functional images). After the normalization procedure, functional images were spatially smoothed with a 6 mm full-width-half-maximum isotropic Gaussian kernel to compensate for residual anatomical variability and to permit application of Gaussian random field theory for statistical inference.^48^ Each pre-processed functional volume was individually inspected for oddities before statistical analyses.

In addition, the structural T_1_-weighted image for each stroke patient was converted into a 3D image of the lesion in standard MNI space following automated procedures described in Seghier et al.^49^ These binary lesion images were used here to delineate the lesions, to estimate lesion volume and to generate lesion overlap maps.

### First level statistical analysis

In the first level statistical analysis, each pre-processed functional volume was entered into a subject specific fixed-effect analysis using the general linear model.^50^ All stimulus onset times (for all tasks) were modelled as single events,^51^ with only the correct response trials as regressors of interest along with three extra regressors of no interest: instructions, incorrect responses and missing responses. Stimulus functions were then convolved with a canonical hemodynamic response function. To exclude low-frequency confounds, the data were high-pass filtered using a set of discrete cosine basis functions with a cut-off period of 128 s. The contrasts of interest compared each of the different tasks (correct trials only) to resting fixation (within session).

### Second level statistical analysis

In the second level statistical analysis, the contrast images from the first level analysis (comparing correct responses against rest) were submitted to a two-way mixed ANOVA with group (patients with LpOp damage and neurologically intact controls) and task (pseudoword reading and repetition) as the factors. There were three contrasts of interest. The first contrast tested for brain regions where activation during both pseudoword tasks was common to patients with LpOp damage and neurologically intact controls (i.e. [1 1 1 1]) and higher than rest in patients with LpOp damage (using inclusive masks [1 0 0 0] and [0 1 0 0]) and neurologically intact controls (using inclusive masks [0 0 1 0] and [0 0 0 1]). This allowed us to identify the regions within the normal system that support accurate behaviour in the patients. In addition, to minimize the possibility that activation in patients with LpOp damage was driven by only one or two outliers, we inclusively masked contrast 1 with a functional overlap map which comprised voxels activated (using a voxel-wise threshold of *P *<* *0.001 uncorrected) in at least four out of seven patients with LpOp damage during pseudoword reading and/or repetition at the first level. The second contrast tested for brain regions where activation during both pseudoword tasks was lower in patients with LpOp damage than neurologically intact controls (i.e. [−1 −1 1 1]) and higher than rest in neurologically intact controls (using inclusive masks [0 0 1 0] and [0 0 0 1]). Reduced activation in spared brain tissue in the patients could be interpreted as a marker of distant lesion effects. Finally, the third contrast tested for brain regions where activation during both pseudoword tasks was higher in patients with LpOp damage than neurologically intact controls (i.e. [1 1 −1 −1]) and higher than rest in patients with LpOp damage (using inclusive masks [1 0 0 0] and [0 1 0 0]). Increased activation in spared brain tissue in the patients could be interpreted as a marker of functional reorganization and adaptive neuroplasticity. As with contrast 1, we inclusively masked contrast 3 with the first level functional overlap map that included activation from at least four of the seven patients with LpOp damage.

The voxel-wise statistical threshold for each of the second level contrasts listed above (including main contrast and inclusive masks) was set at *P *<* *0.05 after family-wise error (FWE) correction for multiple comparisons across the whole brain. Where significant differences between patients with LpOp damage and neurologically intact controls were found (according to all the criteria described above), region-of-interest analyses comparing activation (averaged over all voxels within the cluster) in patients with LpOp damage and patient controls (without LpOp damage) were carried out in IBM SPSS Statistics for Windows, Version 26.0 (IBM Corp., Armonk, NY, USA) to test if the identified effects were lesion-site-dependent, while adjusting for differences in lesion size. This was necessary because the patients with LpOp damage had, on average, larger lesions than the patient controls (*P *=* *0.029; see Table 1 for details). Additional region-of-interest analyses were conducted in SPSS to investigate the pattern of responses across all 13 fMRI tasks (from the two experiments) in neurologically intact controls, and how these task-dependent responses (if any) differed between neurologically intact controls and patients with LpOp damage.

### Data availability

The data that support the findings of this study are available from the senior author (c.j.price@ucl.ac.uk) upon reasonable request.

## Results

### Self-reported recovery of speech production abilities in patients with LpOp damage

**Figure 2:**
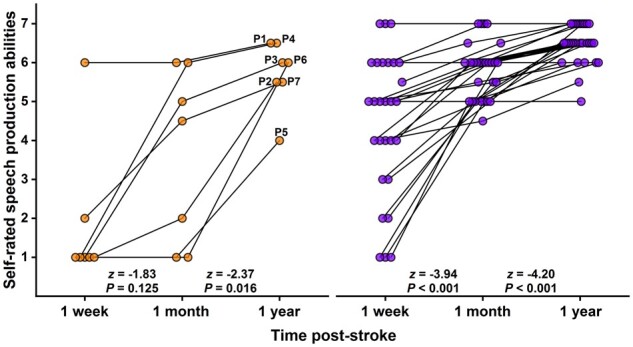
**Self-rated speech production abilities at 1 week, 1 month and 1 year after stroke onset. **As shown in the figure, the patients of interest (with LpOp damage; left panel) and patient controls (without LpOp damage; right panel) rated how their speech production abilities were at 1 week, 1 month and 1 year post-stroke. Each patient was prompted to select one of seven discrete categories on a 7-point in-house ordinal scale using the following question: In relation to your ability to speak at 1 week (or 1 month/1 year) after your stroke, were you? ‘unable to attempt’ (= 1), ‘not speaking at all or using gestures’ (= 2), ‘using gestures but not speaking at all’ (= 3), ‘using only 1 or 2 single words’ (= 4), ‘using a few single words’ (= 5), ‘speaking in short sentences’ (= 6), or ‘speaking normally’ (= 7). In addition, patients were offered the option to select between two consecutive categories: e.g. between ‘speaking in short sentences’ and ‘speaking normally’ (= 6.5). Crucially, patients were instructed to disregard difficulties related to slurred speech. There was a statistically significant positive change in self-rated speech production abilities between 1 week and 1 month post-stroke for patient controls, and between 1 month and 1 year post-stroke for both patient groups.

With the assistance of a speech and language therapist and their carer, each of the patients with LpOp damage retrospectively rated their speech production abilities at 1 week, 1 month and 1 year post-stroke, using an in-house ordinal scale ranging from 1 (= unable to attempt) to 7 (= speaking normally) that is administered to all patients upon entry to the PLORAS study. All seven patients reported that their speech production abilities had (i) been affected by the stroke and (ii) improved from 1 month to 1 year after stroke onset. In contrast, there was greater inter-patient variability in self-reported improvement from 1 week to 1 month post-stroke. Consequently, a Wilcoxon signed-rank test only captured a statistically significant positive change in self-rated speech production abilities between 1 month and 1 year post-stroke (*Z*-score = −2.37, *P *=* *0.016). Despite this self-reported improvement, however, none of the patients with LpOp damage felt that their speech production abilities had completely returned to premorbid levels by 1 year after stroke onset. See Fig. 2 for details.

As a group, the patient controls also reported that their speech production abilities had improved within the first year after stroke onset. However, there were three notable differences relative to the patients with LpOp damage. First, the patient controls’ self-reported improvement in speech production abilities between 1 week and 1 month post-stoke was significant at *P *<* *0.05. Second, unlike the patients of interest, not all the patient controls reported that their speech production abilities had (i) been affected by the stroke; (ii) improved within the first-year post-stroke; and (iii) not returned to premorbid levels by 1 year after stroke onset. Third, the self-rated speech production abilities of the patients with LpOp damage were consistently worse than those of the patient controls across the three time points: 1 week (*U *=* *33.50, *P *=* *0.005), 1 month (*U *=* *38.00, *P *=* *0.009) and 1 year post-stroke (*U *=* *38.00, *P *=* *0.006). See Fig. 2 for details.

### How well can patients with LpOp damage read and repeat pseudowords?

For pseudoword repetition, the normal range of accuracy was between 83% and 100% (mean = 98%). All but 2 of the patients with LpOp damage had normal or near-to-normal accuracy (98%, 98%, 90%, 85% and 78%). The 2 exceptions achieved 73% accuracy (P7) and 68% accuracy (P4). RTs for pseudoword repetition were significantly slower in the patients with LpOp damage than neurologically intact controls [*t*(60) = 2.05, *P *=* *0.044]. Critically, however, there were no significant differences in RTs or accuracy scores between patients with LpOp damage and patient controls (both *P *>* *0.6).

For pseudoword reading, the normal range of accuracy was between 85% and 100% (mean = 98%). All but one of the patients with LpOp damage had normal or near-to-normal accuracy (100%, 95%, 88%, 85%, 83% and 83%). The exception (P6) was correct on 43% of trials. Patient controls attained similar accuracy (on average) as the patients with LpOp damage (*P *=* *0.412). RTs for pseudoword reading were significantly slower in the patients with LpOp damage than neurologically intact controls [*t*(60) = 5.59, *P *<* *0.001], with a (non-significant) trend in the same direction when patients with LpOp damage were compared to patient controls [*t*(33) = 2.02, *P *=* *0.052]. We argue that any group differences in activation that are common to pseudoword reading and repetition cannot be explained by differences in RTs, because RTs during pseudoword repetition were well equated for patients with LpOp damage and patient controls (see above).

Further details of performance differences across the 13 fMRI tasks for both neurologically intact controls and patients with LpOp damage can be found in Table 1 (summary statistics), Supplementary Fig. 2 and Supplementary Table 1.

### Which brain regions support accurate speech production after LpOp damage?

**Figure 3:**
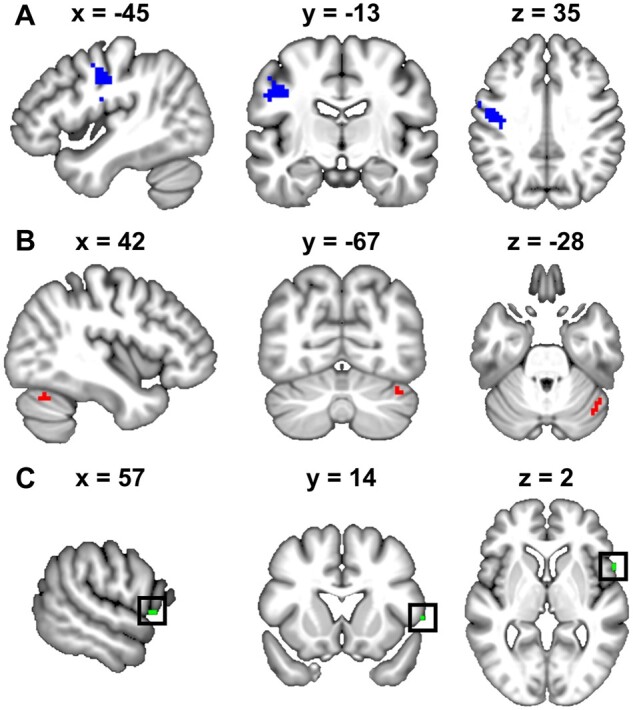
**Brain regions where LpOp damage resulted in reduced or enhanced activation during speech production. **The *top panel* shows the region within left sensorimotor cortex (LS1/M1) where activation was reduced in patients with LpOp damage relative to neurologically intact controls. The *middle panel* shows the region within right cerebellar Crus I (RCrusI) where activation was enhanced in patients with LpOp damage relative to neurologically intact controls. The *bottom panel* shows, surrounded by a square, the region within right ventral pars opercularis (RpOp) where activation was enhanced (albeit at an uncorrected statistical threshold) in patients with LpOp damage relative to neurologically intact controls.

**Figure 4:**
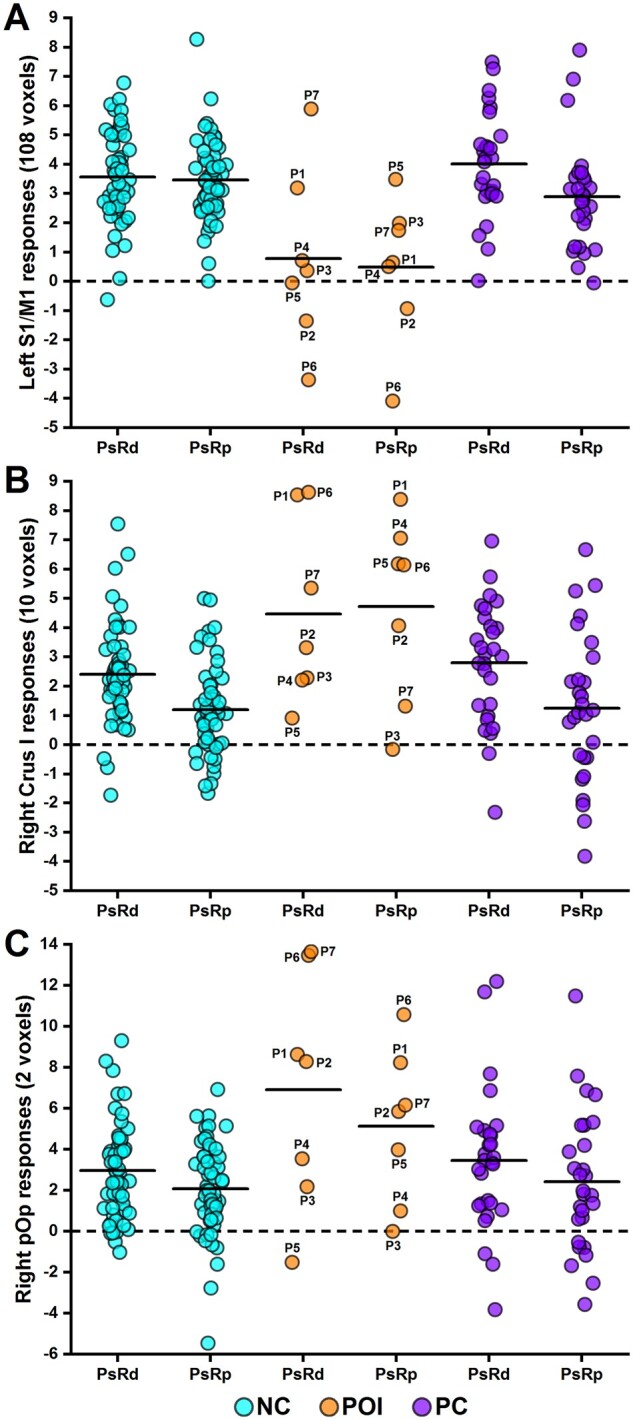
**Inter-subject variability in brain activation during speech production. **The plots show the strength of responses (averaged over voxels) in (**A**) LS1/M1, (**B**) RCrusI and (**C**) RpOp, for each individual participant during accurate pseudoword reading (PsRd) or repetition (PsRp). Thick black lines highlight the corresponding group mean. Dashed black lines signal baseline activation during rest periods (= 0). NC, neurologically intact controls; POI, patients of interest (with LpOp damage); PC, patient controls (without LpOp damage).

During accurate pseudoword production, patients with LpOp damage activated the majority of the intact part of the normal speech production system, including bilateral mid superior temporal cortices, bilateral SMA/pre-SMA, right ventral pars opercularis and right sensorimotor cortex (see Analysis 1 in Table 2 and Supplementary Fig. 3). Unlike neurologically intact controls though, no patient with LpOp damage activated LpOp in the first level analysis. In addition, patients with LpOp damage showed significantly lower activation in a neighbouring left sensorimotor region (LS1/M1; see Fig. 3A) compared to neurologically intact controls (see Analysis 2 in Table 2 and Fig. 4A). LS1/M1 activation was also significantly lower in patients with LpOp damage than patient controls after adjusting for differences in lesion size in the region-of-interest analysis (adjusted means = 0.71 versus 3.43, *P *=* *0.002). The location of LS1/M1 relative to the lesion of each patient with LpOp damage is illustrated in Supplementary Fig. 4.

**Table 2:** MNI coordinates and location of fMRI activation peaks

Brain region	Hemisphere	Coordinates			*Z*-score	*P*_FWE-c_	Extent^a^
		*x*	*y*	*z*			
Analysis 1: common activation in NC and POI during PsRd and PsRp							
S1/M1	R	48	−10	38	Inf	<0.001	148
S1/M1		51	−7	41	Inf	<0.001	
M1		60	−1	17	Inf	<0.001	
M1		51	−7	53	Inf	<0.001	
mSTG	R	60	−25	2	Inf	<0.001	174
mSTG		51	−25	2	Inf	<0.001	
pOp/vPMC		57	11	−1	Inf		
pTr		51	20	−1	7.65	<0.001	
SMA	L	−3	−1	62	Inf		131
SMA/pre-SMA		−3	5	59	Inf		
SMA/pre-SMA	R	3	8	65	Inf		
mSTG	L	−63	−28	5	Inf		90
Heschl’s gyrus		−51	−19	5	Inf	<0.001	
aSTG		−51	8	−13	Inf	<0.001	
Crus I	R	39	−67	−25	Inf	<0.001	51
Crus I		42	−64	−28	Inf		
Analysis 2: NC > POI during PsRd and PsRp							
S1/M1	L	−45	−13	35	4.94	0.016	108
Analysis 3: POI > NC during PsRd and PsRp							
aMFG	L	−36	44	20	5.12	0.007	3^b^
Crus I	R	42	−67	−28	4.75	0.036	10

a, anterior; m, mid; M1, primary motor cortex; MFG, middle frontal gyrus; NC, neurologically intact controls; POI, patients of interest with LpOp damage; PsRd, pseudoword reading; PsRp, pseudoword repetition; PMC, premotor cortex; pOp, pars opercularis of the inferior frontal gyrus; pTr, pars triangularis of the inferior frontal gyrus; S1, primary somatosensory cortex; STG, superior temporal gyrus; SMA, supplementary motor area; pre-SMA, pre-supplementary motor area; v, ventral.

a The extent of the effects was defined after lowering the voxel-wise threshold to *P *<* *0.001 uncorrected.

b Effects comprising less than five contiguous voxels were not pursued any further.

The effect of most interest was that patients with LpOp damage showed increased activation within right cerebellar Crus I (RCrusI; see Fig. 3B) compared to neurologically intact controls (see Analysis 3 in Table 2 and Fig. 4B). RCrusI activation was also significantly higher in patients with LpOp damage than patient controls after adjusting for differences in lesion size in the region-of-interest analysis (adjusted means = 4.33 versus 2.10, *P *=* *0.025).

Consistent with Rosen et al.^31^ and Blank et al.,^32^ we also found that activation (at *P *<* *0.001 uncorrected) was higher in patients with LpOp damage compared to neurologically intact controls in the right ventral pars opercularis (RpOp, *Z*-score = 4.09 at [57, 14, 2], *K* = 2 voxels; see Figs. 3C and 4C). In contrast, the region-of-interest analysis indicated that the difference in RpOp activation between patients with LpOp damage and patient controls was not significant after adjusting for differences in lesion size (adjusted means = 5.05 versus 3.18, *P *=* *0.203).

At the individual subject level, all but one (i.e. P3) of the seven patients with LpOp damage showed increased activation during pseudoword production in either RCrusI, RpOp or both, compared to neurologically intact controls; see Fig. 4 and Supplementary Table 4 for details.

### What is the function of the brain regions where activation decreased/increased in patients with LpOp damage?

The focus of the results above has been on two tasks (i.e. reading and repeating pseudowords) that were expected to robustly engage LpOp on the basis of prior findings. In addition, all our participants completed 11 other tasks (see Materials and methods for details). By examining the pattern of responses across all 13 tasks (from the two experiments), we can infer the type of function that LS1/M1, RCrusI and RpOp subserve in the neurologically intact brain. These region-of-interest analyses (i.e. activation averaged over all voxels within the cluster) were carried out in SPSS.

**Figure 5:**
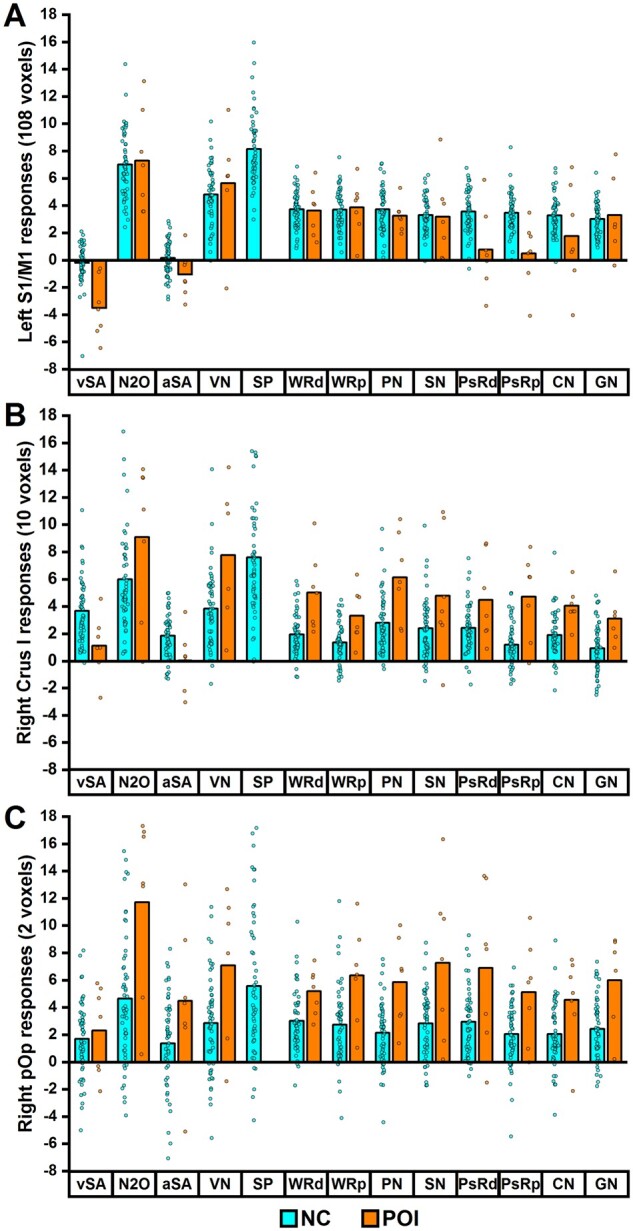
**Response profile of distinct brain regions. **The plots show the subject-level (circles) and group-level (bars = mean) activation response in (**A**) LS1/M1, (**B**) RCrusI and (**C**) RpOp, for both 55 neurologically intact controls (NC) and seven patients with LpOp damage (POI) during each of 13 fMRI tasks (vSA to SP = Experiment 1; WRd to GN = Experiment 2). During some tasks, the activation response in RCrusI for one control (N2O = 21.4 and SP = 27.9) and in RpOp for another (aSA = −10.8) fell outside the range of values displayed. Mean activation for each task in each group was calculated after excluding data points from tasks when a participant achieved less than 40% accuracy across trials. In total, five (out of 804) data points were excluded, three from patients with LpOp damage (1 for VN and 2 for SP) and two from neurologically intact controls (1 for aSA and 1 for SP). For statistical analyses, these excluded data points were replaced with the mean activation for that task for that group (mean imputation never occurred for more than a single data point in any given task per group), except for SP in patients with LpOp damage (not shown on plots) due to the high number (3/7) of excluded data points. aSA, auditory semantic associations; CN, naming colour of meaningless pattern; GN, naming gender of voice humming meaningless rhythm; N2O, naming two objects from picture; PN, naming one object from picture; PsRd, pseudoword reading; PsRp, pseudoword repetition; SN, naming one object from sound; SP, sentence production; VN, verb naming; vSA, visual semantic associations; WRd, word reading; WRp, word repetition. The order of presentation in the scanner was slightly different: vSA, N2O, VN, SP, aSA, WRd, WRp, PN, CN, SN, PsRd, PsRp and GN.

LS1/M1 activation in neurologically intact controls occurred during the 11 speech production tasks (3 in Experiment 1 and 8 in Experiment 2) but not during the two silent semantic matching tasks (Experiment 1), with a statistically significant difference (*P *<* *0.05 for all comparisons) between each speech production task and each sematic matching task (see Fig. 5A). Effects of stimulus modality, semantic or phonological processing during speech production (Experiment 2) did not influence LS1/M1 activation. This contrasts with the task-specific response observed in LS1/M1 in patients with LpOp damage, who showed lower than normal LS1/M1 activation during pseudoword reading/repetition (and less markedly during colour naming) but not during other speech production tasks (see Fig. 5A). Accordingly, a group by task interaction (*P *<* *0.001) indicated that the reduction in LS1/M1 activation affected pseudoword reading/repetition more than word reading/repetition. The responses of LS1/M1 in each patient with LpOp damage during each fMRI task can be found in Supplementary Table 5.

RCrusI activation in neurologically intact controls occurred during all 13 tasks (across Experiments 1 and 2), irrespective of the demands on speech production, phonological processing, semantic processing or perceptual processing (see Fig. 5B). In patients with LpOp damage, RCrusI activation was increased, compared to neurologically intact controls, during all 11 speech production tasks across Experiments 1 and 2 (*P *<* *0.05 for all comparisons apart from the task of naming 2 objects in Experiment 1 where *P *=* *0.070). In contrast, during the two silent semantic matching tasks (Experiment 1), RCrusI activation was reduced, rather than enhanced, in patients with LpOp damage compared to neurologically intact controls (*P *<* *0.05 for both comparisons). This resulted in a significant interaction (*P *=* *0.001) between group (patients with LpOp damage > neurologically intact controls) and task (naming pictures of 2 objects > semantic decisions on pictures of 2 objects); see Fig. 5B.

RpOp activation in neurologically intact controls occurred during all 13 tasks (across Experiments 1 and 2), with stronger responses during all speech production tasks than silent semantic matching tasks (see Fig. 5C); but with no effect of stimulus modality, semantic or phonological processing during speech production (Experiment 2). In patients with LpOp damage, RpOp activation was higher than that in neurologically intact controls across all 13 tasks (see Fig. 5C), and this enhancement was significantly greater for naming pictures of 2 objects than semantic decisions on pictures of 2 objects (Experiment 1) as evidenced by a significant group by task interaction (*P *=* *0.001).

### Is increased RCrusI activation the consequence of LpOp damage or decreased LS1/M1 activation?

**Figure 6:**
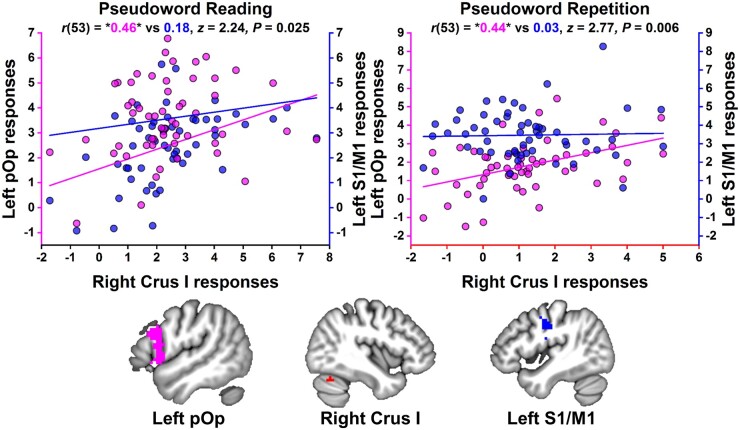
**Comparison of co-activation strength in neurologically intact controls.** The plots show that in 55 neurologically intact controls the relationship between LpOp activation and RCrusI activation (in magenta) was stronger than that between LS1/M1 activation and RCrusI activation (in blue) during both pseudoword reading and repetition. Correlation coefficients surrounded by asterisks were statistically significant at *P *<* *0.05. The left pOp region uniquely comprised voxels, within an anatomically defined mask of left pOp, that were activated during both pseudoword reading and repetition in neurologically intact controls at *P *<* *0.001 uncorrected.

By focussing on the fMRI data from neurologically intact controls, we found that, during pseudoword reading and repetition, RCrusI activation co-varied positively with activation in LpOp (where the patients of interest had structural damage) but not LS1/M1 (where the patients of interest had lower activation than controls, but no detectable structural damage). The strength of covariance with RCrusI was also significantly higher for LpOp than LS1/M1 (see Fig. 6).

Within LpOp, covariance with RCrusI activation during pseudoword reading and repetition peaked in two distinct parts of the pars opercularis (pOp). The more dorsal pOp peak was located at [−54, 14, 17] (*Z*-score = 5.14, *P *=* *0.007 FWE-corrected across the whole brain, *K* = 4 voxels). The more ventral pOp peak was located at [−54, 17, −1] (*Z*-score = 4.82, *P* = 0.032 FWE-corrected across the whole brain, *K* = 2 voxels); see Supplementary Fig. 5A. There were no significant differences in the strength with which these pOp regions co-varied with RCrusI (see Supplementary Fig. 5B). No other speech production region in the left frontal lobe (e.g. primary motor cortex or ventrolateral premotor cortex) co-varied significantly with RCrusI during pseudoword reading and repetition.

When we examined how the responses in the dorsal and ventral parts of pOp were influenced by the type of speech production task (Experiment 2), we found that the dorsal pOp region was sensitive to the demands on phonological processing during speech production (i.e. greater in the presence than the absence of phonological content; *Z*-score = 4.94 at [−54, 14, 17], *P *=* *0.016 FWE-corrected across the whole brain). In contrast, the ventral pOp region did not track the demands on perceptual, semantic or phonological processing during speech production (i.e. no significant main effect of any factor, as observed in RCrusI); see Supplementary Fig. 5C.

### Is increased RpOp activation a result of dis-inhibition following LpOp damage?

There was no evidence that the enhanced activation within RpOp in patients with LpOp damage could be explained by dis-inhibition (i.e. an inhibitory relationship between LpOp and RpOp that was lost following LpOp damage). To the contrary, we found that in neurologically intact controls, there was positive (not negative) covariance between the responses of the RpOp region that showed the highest activation in patients with LpOp damage (at coordinates [57, 14, 2]) and responses of the homologous LpOp region (at coordinates [−57, 14, 2]). This was observed during both pseudoword reading [*r*(53) = 0.34, *P *=* *0.011] and pseudoword repetition [*r*(53) = 0.43, *P *=* *0.001].

## Discussion

The goal of this study was to investigate the brain activation patterns that underlie accurate overt speech production in chronic stroke patients with relatively circumscribed left frontal lobe damage involving the posterior part of Broca’s area: left pars opercularis (LpOp). All seven patients with LpOp damage included in this study reported, using an in-house ordinal scale, that their speech production abilities had improved in the first-year post-stroke compared to how they were initially after stroke onset. By focussing on accurate overt speech responses, our study has yielded the following three novel observations: (i) activation in a specific region within the left sensorimotor cortex (LS1/M1, neighbouring the left posterior frontal lesion) was significantly lower in patients with LpOp damage relative to neurologically intact controls during pseudoword production but not other tasks, suggesting that the relationship between LpOp and LS1/M1 is task-sensitive; (ii) activation in a specific part of right cerebellar Crus I (RCrusI) was significantly higher in patients with LpOp damage relative to both neurologically intact controls and patients without LpOp damage, suggesting that RCrusI may play a particularly important role in compensating for the effect of LpOp damage on speech production; and (iii) higher activation in the right ventral pars opercularis (RpOp) co-varied with higher activation in its left-hemisphere homologue in neurologically intact controls, suggesting that increased RpOp activation following LpOp damage cannot be explained in terms of dis-inhibition.

Below we discuss these findings in light of prior literature to highlight the novel insights they provide for understanding how accurate speech production is achieved after damage to the opercular part of Broca’s area and the functional anatomy of speech production.

### Task-specific reduction of LS1/M1 activation after damage to the opercular part of Broca’s area

Although patients with LpOp damage recruited most of the intact part of the speech production system as normal, we identified a region within the left sensorimotor cortex (LS1/M1, neighbouring the left posterior frontal lesion) that showed reduced activation relative to neurologically intact controls. Overall, the response pattern of this LS1/M1 region across tasks was similar for neurologically intact controls and patients with LpOp damage. In both groups, LS1/M1 activation was greater during overt speech production than silent semantic matching, with activation during silent semantic matching not raising above resting baseline levels. This is consistent with a role for LS1/M1 in the sensorimotor control of speech. Interestingly, reduced LS1/M1 activation in patients with LpOp damage compared to neurologically intact controls was task-specific, affecting pseudoword reading/repetition but not other tasks. Although the current dataset does not allow us to unequivocally elucidate the underlying cause of such a response profile, in what follows we offer three tentative explanations to motivate further research, with the one we favour considered last.

First, the reduction in LS1/M1 activation during pseudoword reading and repetition might be the consequence of altered cerebral perfusion in tissue neighbouring the lesion but without evident structural damage.^52^^,^^53^ We argue this is unlikely to be the case because it would not explain why LS1/M1 responded normally during speech production tasks that did not involve pseudowords. Second, the task-specific reduction in LS1/M1 activation might be the consequence of propagation of tissue damage into the ischaemic penumbra.^54^ Visual inspection of the T_1_-weighted images of the patients with LpOp damage does raise questions about whether or not LS1/M1 activation in P6 fell within compromised tissue (see Supplementary Fig. 4). However, Supplementary Table 5 shows that LS1/M1 in P6 was, at the very least, not entirely dysfunctional given the response observed during other fMRI tasks. Moreover, when P6 was removed from the analysis, LS1/M1 activation during pseudoword production remained significantly lower in the patients with LpOp damage than neurologically intact controls (means = 1.35 versus 3.52, *P *<* *0.001) and patient controls (means = 1.35 versus 3.45, *P *=* *0.007). Third, the task-specific reduction in LS1/M1 activation might be the consequence of a loss of excitatory inputs from LpOp to LS1/M1 during pseudoword production following LpOp damage. In other words, this explanation suggests that the relationship between LpOp and LS1/M1 is task-sensitive, consistent with the notion of dynamic diaschisis where abnormal evoked responses in a structurally intact region depend on task-relevant neuronal interactions with a damaged region.^55^

Finally, the fact that patients with LpOp damage were able to produce accurate overt speech responses during pseudoword reading/repetition indicates that reduced LS1/M1 activation was either sufficient for task performance or these patients were using alternative neural pathways to bypass LS1/M1. Future effective connectivity studies are needed to investigate these hypotheses directly, by examining, for example, how LpOp and LS1/M1 influence one another and how these influences depend on the task (e.g. pseudoword versus word production).

### The role of RCrusI in accurate speech production after damage to the opercular part of Broca’s area

Unlike the two previous functional imaging studies that investigated overt speech production in chronic stroke patients with left frontal lobe damage involving Broca’s area,^31^^,^^32^ we found that activation in a specific part of the right posterolateral cerebellum (i.e. RCrusI) was increased in patients with LpOp damage compared to neurologically intact controls and patients without LpOp damage. Since speech production was more laborious than normal in patients with LpOp damage as indicated by slower and more error-prone responses, increased RCrusI activation during accurate speech production suggests that the right cerebellar circuit was working harder than normal to offset the impact of brain damage. This complements the findings of the study by Metter et al.^56^ who showed that aphasic speech is worse when damage in and around Broca’s area is accompanied by reduced metabolism in the right lateral cerebellum. The compensatory potential of right cerebellar regions has also been demonstrated by previous studies where transcranial direct current stimulation over the right posterolateral cerebellum was found to (i) improve speech fluency in neurologically intact individuals and (ii) boost the recovery of speech production abilities in stroke patients with heterogeneous left-hemisphere lesion locations.^57–60^ Above and beyond these previous findings, our results localize a specific target region within the right posterolateral cerebellum (i.e. RCrusI) for future non-invasive neurostimulation therapeutic studies and suggest that stimulating this region could be a promising approach to enhancing the speech production abilities of patients with damage to the opercular part of Broca’s area.

Importantly, the RCrusI region we have identified does not correspond to the areas of the cerebellum in bilateral lobules I–VI and VIII that have previously been associated with sensorimotor processing,^61^^,^^62^ including speech articulation.^63^ Instead, RCrusI has been implicated in higher order cognitive/language processing.^61^^,^^62^^,^^64–66^ Indeed, our fMRI data from neurologically intact controls show that this right cerebellar region was activated during all conditions, irrespective of the demands on speech production, phonological processing, semantic processing or perceptual processing. We interpret such a response pattern across tasks as being potentially consistent with a role for RCrusI in domain-general cognitive control. Further light on the role of RCrusI in cognition is shed by D’Mello et al.^67^ who (i) revealed a hierarchical organization of cognitive control in the right (and left) posterolateral cerebellum and (ii) associated RCrusI with the ability to integrate abstract and concrete processing to optimize current and future behaviour in a context-sensitive manner. In the speech domain specifically, Runnqvist et al.^68^ maximized the demands on self-monitoring during speech production by priming the production of speech errors in the context of a speeded task. At the same time, they transiently disrupted right cerebellar Crus I/II with repetitive transcranial magnetic stimulation (rTMS), which resulted in increased error rates, slowed RTs (for correct trials) and reduced learning relative to rTMS over the homologous left cerebellar region. Thus, RCrusI might contribute to accurate speech production following damage to the opercular part of Broca’s area through internal modelling of upcoming speech and error-based learning, potentially in close interaction with multiple cerebral regions, including those within the left-lateralised perisylvian speech network, thanks to its rich structural/functional connectivity.^69–74^ Future studies are now required to investigate how exactly RCrusI contributes to accurate speech production after damage to the opercular part of Broca’s area.

Does increased RCrusI activation during accurate speech production reflect a compensatory mechanism triggered by damage to the opercular part of Broca’s area? Although further research is needed, four lines of evidence (three from our study and one from previous work) support an affirmative answer. First, increased RCrusI activation was observed in patients with, but not without, LpOp damage, indicating a lesion-site-dependent effect. Second, in neurologically intact controls, higher RCrusI activation co-varied with higher activation in LpOp but not LS1/M1 and the specificity of this inter-regional coupling was confirmed by a significant difference in the strength of covariance between RCrusI and LpOp versus RCrusI and LS1/M1. Third, no other speech production region within the left frontal lobe (apart from LpOp) exhibited significant positive covariance with RCrusI activation in neurologically intact controls. Fourth, prior reports have shown that (i) RCrusI is co-activated with LpOp but not, for example, primary motor cortex during task performance and (ii) a transient reduction in blood flow and metabolism in the right lateral cerebellum can result from damage in and around Broca’s area.^75–78^

### The role of RpOp in accurate speech production after damage to the opercular part of Broca’s area

The two previous functional imaging studies of overt speech production in chronic stroke patients with left frontal lobe damage involving Broca’s area, both reported increased activation within the right posterior inferior frontal gyrus (RpIFG) relative to neurologically intact controls.^31^^,^^32^ We have replicated this finding using a much larger group of neurologically intact controls and two different speech production tasks (i.e. pseudoword reading and repetition) selected on the basis of current knowledge regarding the role of LpOp in speech production. In terms of the precise anatomical localization of increased activation within RpIFG, our results are most consistent with those of Blank et al.^32^ who localized it to the right pars opercularis (RpOp). Critically, Rosen et al.^31^ and Blank et al.^32^ speculated that increased RpIFG activation might have been the consequence of release from an inhibitory relationship with Broca’s area (i.e. dis-inhibition). Our findings are not consistent with this hypothesis because we found positive, not negative, covariance between RpOp activation and LpOp activation in neurologically intact controls. Moreover, neither Rosen et al.^31^ nor Blank et al.^32^ could dissociate brain responses elicited by accurate versus inaccurate overt speech production due to the low temporal resolution of PET imaging. By focussing our analyses on corrects trials only, we have therefore provided novel evidence that increased RpOp activation following LpOp damage supports accurate overt speech production. This is in agreement with a study of neurologically intact participants by Hartwigsen et al.^38^ who reported that faster overt speech responses (i.e. more efficient performance) following disruptive rTMS over LpOp were associated with increased effective connectivity from RpOp to LpOp during pseudoword reading and repetition. The importance of RpOp for speech production after left-hemisphere damage has also been highlighted by Naeser et al.^79^ who showed that disruptive rTMS to RpOp slowed picture naming in a group of right-handed chronic stroke patients with heterogeneous left-hemisphere lesion locations.

Regarding the role of RpOp in accurate speech production, we examined its response over a wide range of tasks. This indicated that, in neurologically intact controls, RpOp (i) responded during all 13 tasks but with greater strength during overt speech production than silent semantic matching (Experiment 1) and (ii) was insensitive to the demands on perceptual, semantic or phonological processing during speech production (Experiment 2). We propose that such a response profile is consistent with a role for RpOp in domain-general cognitive control that is enhanced by, but not specific to, speech production. Further light on the role of RpOp is shed by prior studies that have largely associated this area with a specific component of cognitive control, namely response inhibition that is needed to suppress errors.^80–84^ In our study, RpOp activation was highest (i) for complex overt speech responses during speech production compared to simple button press responses during semantic matching (where the demands on response inhibition are expected to be higher versus lower, respectively) and (ii) after LpOp damage compared to other left-hemisphere lesion sites. Plausibly, this is because bilateral inferior frontal regions support response inhibition,^85^ with the right being able to compensate for loss of the left. Future studies are required to investigate how exactly RpOp contributes to accurate speech production after damage to the opercular part of Broca’s area.

## Conclusion

We have shown that patients with damage to the opercular part of Broca’s area are able to recruit most of the intact part of the normal system to support accurate overt speech production. Within this system, activation in two specific regions within the right cerebellar Crus I (RCrusI) and right pars opercularis (RpOp) was increased following LpOp damage compared to controls. We have discussed how these regions might help to compensate for the effect of damage to the opercular part of Broca’s area. Our findings thus motivate and guide future studies with larger patient numbers to investigate: (i) how exactly RCrusI and RpOp support accurate speech production after damage to the opercular part of Broca’s area; (ii) how RCrusI and RpOp activation changes over time as a function of speech production recovery after damage to the opercular part of Broca’s area; (iii) what lesion and non-lesion factors explain inter-patient variability in RCrusI and RpOp activation after damage to the opercular part of Broca’s area; and (iv) whether non-invasive neurostimulation to RCrusI and/or RpOp boosts speech production recovery in patients with, versus without, damage to the opercular part of Broca’s area.

## Supplementary material

Supplementary material is available at *Brain Communications* online.

## Supplementary Material

fcab230_Supplementary_Data

## Appendix

PLORAS Team members contributed to the acquisition and analysis of behavioural and neuroimaging data. They include: Louise Lim, Rachel Bruce, Hayley Woodgate, Sophie Roberts, Kate Ledingham, Shamima Khan and Storm Anderson.

## Abbreviations

fMRI =: functional magnetic resonance imaging
LpOp =: left pars opercularis
LS1/M1 =: left sensorimotor cortex
RCrusI =: right cerebellar Crus I
RpIFG =: right posterior inferior frontal gyrus
RpOp =: right pars opercularis
rTMS =: repetitive transcranial magnetic stimulation
SMA =: supplementary motor area
