# Supplementary material for: Brain regions that support accurate speech production after damage to Broca’s area
Source: Brain Commun. 2021 Oct 1;3(4):fcab230. doi: 10.1093/braincomms/fcab230 (PMC8523882; doi:10.1093/braincomms/fcab230)
Supplement: fcab230_Supplementary_Data [file fcab230_supplementary_data.pdf]

Supplementary Table 1. Accuracy for fMRI tasks

	P1	P2	P3	P4	P5	P6	P7	POI	PC	NC
**vSA**	100.0	85.0	90.0	70.0	95.0	100.0	90.0	90.0 (±10.4)	90.0 (±12.3)	95.6 (±7.1)
**N2O**	70.0	90.0	90.0	55.0	70.0	75.0	65.0	73.6 (±12.8)	86.1 (±11.7)	93.1 (±5.3)
**aSA**	95.0	95.0	90.0	80.0	90.0	100.0	80.0	90.0 (±7.6)	88.6 (±10.1)	94.6 (±9.8)
**VN**	95.0	100.0	100.0	15.0	90.0	85.0	80.0	80.7 (±29.9)	88.0 (±14.7)	96.4 (±6.6)
**SP**	40.0	75.0	80.0	---	5.0	40.0	15.0	42.5 (±30.5)	58.0 (±26.7)	89.0 (±12.6)
**WRd**	100.0	100.0	100.0	97.5	100.0	100.0	92.5	98.6 (±2.8)	97.5 (±4.2)	99.9 (±0.5)
**WRp**	94.9	100.0	95.0	97.5	100.0	97.5	97.5	97.5 (±2.1)	94.5 (±8.0)	99.5 (±1.3)
**PN**	90.0	92.5	97.5	65.0	82.5	80.0	72.5	82.9 (±11.5)	90.0 (±10.2)	96.8 (±3.1)
**SN**	77.5	50.0	85.0	42.5	60.0	60.0	55.0	61.4 (±15.0)	69.1 (±20.1)	92.9 (±10.2)
**PsRd**	95.0	82.5	100.0	85.0	82.5	42.5	87.5	82.1 (±18.7)	87.7 (±15.0)	98.1 (±3.3)
**PsRp**	97.5	90.0	97.5	67.5	85.0	77.5	72.5	83.9 (±11.9)	84.6 (±14.4)	97.7 (±4.5)
**CN**	100.0	100.0	100.0	90.0	55.0	100.0	95.0	91.4 (±16.5)	95.2 (±5.8)	98.5 (±4.2)
**GN**	72.5	95.0	100.0	---	95.0	90.0	67.5	86.7 (±13.4)	93.3 (±14.1)	98.0 (±5.9)

The first seven columns of the table show the accuracy score (= % correct) for each patient of interest with LpOp damage on each fMRI task. Increasingly darker shades of grey highlight accuracy scores that were ≥1, ≥2 or ≥3 standard deviations below the mean accuracy score for that particular task in neurologically-intact controls (= NC). The last three columns provide the mean accuracy score (± 1 standard deviation) for patients of interest (= POI), patient controls (= PC) and neurologically-intact controls (= NC). vSA = visual semantic associations; N2O = naming two objects from picture; aSA = auditory semantic associations; VN = verb naming; SP = sentence production; WRd = word reading; WRp = word repetition; PN = naming one object from picture; SN = naming one object from sound; PsRd = pseudoword reading; PsRp = pseudoword repetition; CN = naming colour of meaningless pattern; GN = naming gender of voice humming meaningless rhythm.

Supplementary Table 2. Speech and language assessment scores

	P1	P2	P3	P4	P5	P6	P7	POI	PC
**Months between stroke and CAT**	61.1	52.0	168.6	133.0	58.2	9.6	99.9	83.2 (±54.1)	68.5 (±69.5)
**Months between CAT and fMRI**	12.6	27.5	0.4*	4.2	0.0	69.6	6.0	17.2 (±25.0)	29.3 (±35.8)
**SemMatch**	51.0	60.0	60.0	51.0	60.0	60.0	60.0	57.4 (±4.4)	59.0 (±2.8)
**SpkLangComp**	74.0	59.0	63.0	57.0	62.0	65.0	60.0	62.9 (±5.6)	61.6 (±4.7)
**WrittLangComp**	65.0	58.0	73.0	57.0	62.0	62.0	57.0	62.0 (±5.7)	64.0 (±4.7)
**Naming**	64.0	60.0	68.0	63.0	59.0	65.0	56.0	62.1 (±4.1)	69.0 (±4.7)
**Repetition**	60.0	62.0	66.0	59.0	59.0	62.0	56.0	60.6 (±3.2)	59.1 (±5.6)
**Reading**	66.0	60.0	71.0	66.0	64.0	58.0	53.0	62.6 (±6.0)	65.5 (±4.9)
**Writing**	62.0	65.0	69.0	61.0	62.0	65.0	69.0	64.7 (±3.3)	64.5 (±3.8)
**SpkPicDesc**	66.0	64.0	64.0	59.0	58.0	58.0	52.0	60.1 (±4.8)	65.5 (±4.3)
**WrittPicDesc**	68.0	67.0	70.0	58.0	62.0	62.0	69.0	65.1 (±4.5)	67.8† (±6.9)

The overall level of performance of each patient of interest (with LpOp damage) across a set of speech and language tasks from the Comprehensive Aphasia Test (CAT) is summarized in the form of T-scores (i.e. standardized scores with a mean of 50 and a standard deviation of 10). T-scores highlighted in red signal impaired performance. Summary statistics (mean ± 1 standard deviation) for patients of interest (POI) and patient controls (PC) are provided in the last two columns. SemMatch = semantic picture-to-picture matching; SpkLangComp = spoken language comprehension, including comprehension of spoken words, sentences and paragraphs; WrittLangComp = written language comprehension, including comprehension of written words and sentences; Naming = spoken picture naming of objects and actions in addition to word fluency; Repetition = repeating aloud words, complex words, pseudowords, digit strings and sentences; Reading = reading aloud words, complex words, function words and pseudowords; Writing = copying text, written picture naming and writing to dictation; SpkPicDesc = spoken picture description; WrittPicDesc = written picture description.

* P3 was the only patient of interest whose speech and language assessment session took place (12 days) after, rather than before, the fMRI session

† One patient control did not complete the written picture description task from the CAT

Supplementary Table 3. Statistical analysis of in-scanner behavioural data

**Effect**	***df***	***F***	***p*-value**
		**Accuracy**	
NC: Phon x Sem	1, 54	26.20	< 0.001
Group	1, 60	99.25	< 0.001
Group x Phon x Sem	1, 60	41.68	< 0.001
Group x Phon x Mod	1, 60	13.95	< 0.001
Group x Sem x Mod	1, 60	6.19	0.016
		**Response times**	
NC: Modality	1, 54	578.03	< 0.001
NC: Phon x Sem	1, 54	118.69	< 0.001
NC: Phon x Mod	1, 54	10.50	0.002
NC: Sem x Mod	1, 54	17.41	< 0.001
NC: Phon x Sem x Mod	1, 54	5.78	0.020
Group	1, 60	36.74	< 0.001
Group x Phon	1, 60	6.50	0.013
Group x Phon x Mod	1, 60	5.30	0.025

Only significant effects involving the “group” factor are reported for the mixed ANOVAs (Experiment 2) as these were the focus of the analysis. NC = neurologically-intact controls; Group = neurologically-intact controls and patients of interest with LpOp damage.

Supplementary Table 4. Increased brain activation in patients with LpOp damage

	Right Crus I		Right pOp	
	**PsRd**	**PsRp**	**PsRd**	**PsRp**
**P1**	3.65	4.71	2.47	2.73
**P2**	0.54	1.89	2.31	1.68
**P3**	-0.07	-0.88	-0.33	-0.91
**P4**	-0.12	3.85	0.26	-0.47
**P5**	-0.89	3.28	-1.93	0.85
**P6**	3.71	3.25	4.57	3.77
**P7**	1.76	0.09	4.64	1.82

For each patient of interest with LpOp damage, activation in right cerebellar Crus I and right pOp is expressed in terms of number of standard deviations above or below the mean activation (= Z-scores) in that particular brain region in neurologically-intact controls during pseudoword reading (PsRd) or pseudoword repetition (PsRp). Increasingly darker shades of grey highlight brain responses that were ≥1, ≥2 or ≥3 standard deviations above the mean.

Supplementary Table 5. LS1/M1 activation in patients with LpOp damage across 13 fMRI tasks

	P1	P2	P3	P4	P5	P6	P7
**vSA**	-0.87	-4.81	-0.60	-6.45	-3.59	-5.19	-3.09
**N2O**	6.96	11.03	7.96	3.59	4.79	3.59	13.14
**aSA**	-1.60	-0.19	1.84	-1.54	-2.35	-0.35	-3.25
**VN**	7.37	5.14	6.15	---	-2.06	6.21	11.02
**SP**	11.80	12.04	6.04	---	---	-5.02	---
**WRd**	4.09	2.54	5.00	1.30	6.42	1.84	4.16
**WRp**	4.86	3.52	4.38	0.31	4.58	2.67	6.71
**PN**	5.30	3.56	3.01	3.10	3.56	2.28	1.94
**SN**	2.80	0.17	4.47	1.65	0.08	4.15	8.86
**PsRd**	3.19	-1.34	0.38	0.71	-0.05	-3.35	5.90
**PsRp**	0.65	-0.92	1.99	0.51	3.49	-4.09	1.75
**CN**	3.33	0.80	5.52	0.60	-4.02	-0.74	6.83
**GN**	1.41	2.42	7.76	---	-0.38	2.64	6.00

Activation in the left sensorimotor (LS1/M1) region, where LpOp damage resulted in reduced activation during pseudoword production, is shown for each patient of interest (with LpOp damage) and fMRI task. Positive and negative values (in arbitrary units) indicate brain responses that were above or below resting baseline levels, respectively. P4 did not complete the SP and GN tasks. The responses of LS1/M1 during VN for P4 and SP for P5 and P7 are not shown due to poor performance (i.e. < 40% accuracy).

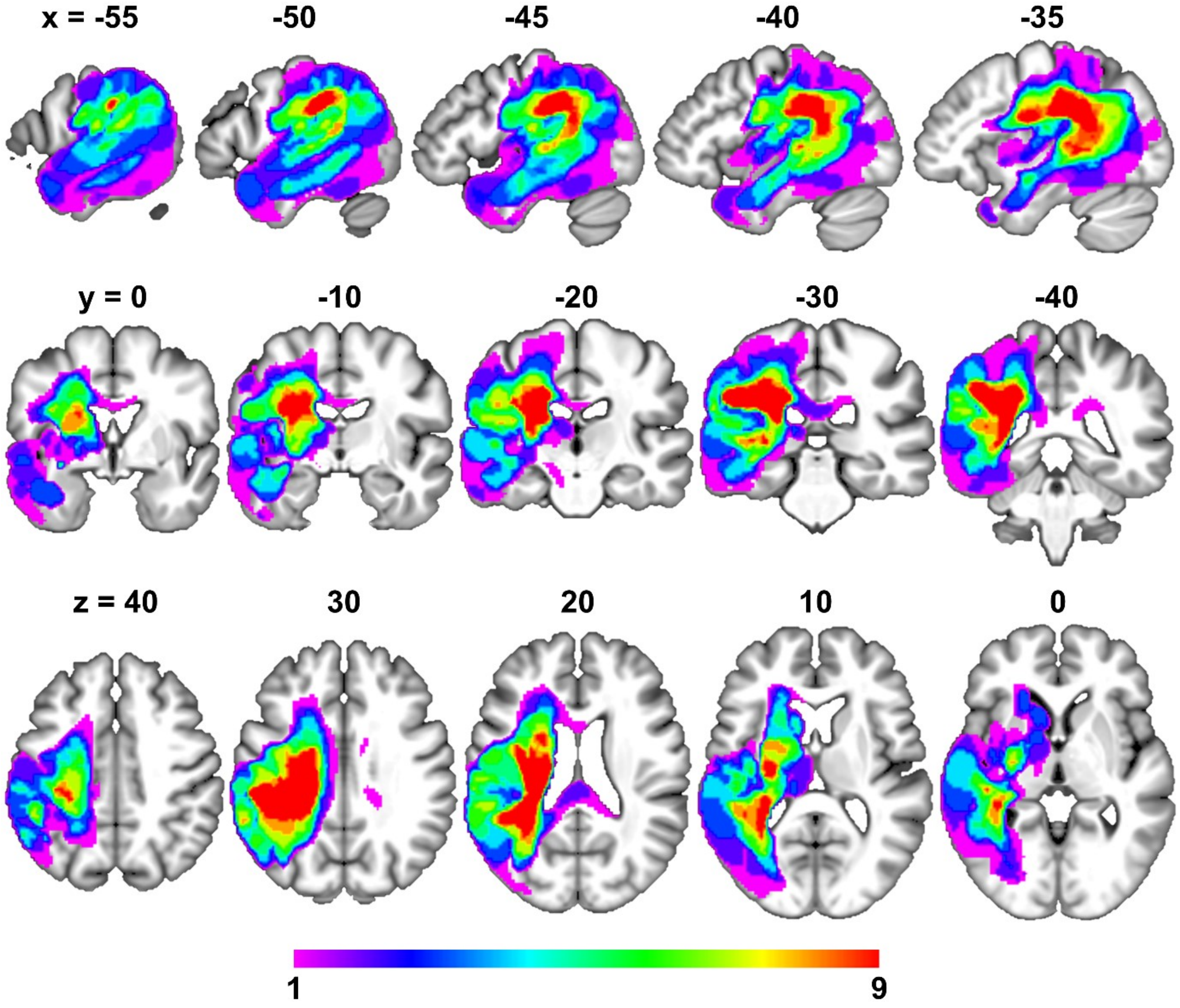

Supplementary Figure 1. Lesion overlap map of 28 patients with unilateral left-hemisphere strokes sparing LpOp. Overlaid on a brain template, the map shows the lesions of 28 stroke patients who all preserved the left pars opercularis (i.e. patient controls). The colour scale indicates the number of overlapping lesions at each given voxel. The brain regions most frequently damaged in these patient controls were the left rostroventral supramarginal gyrus and the underlying white matter in the vicinity of the arcuate fasciculus.

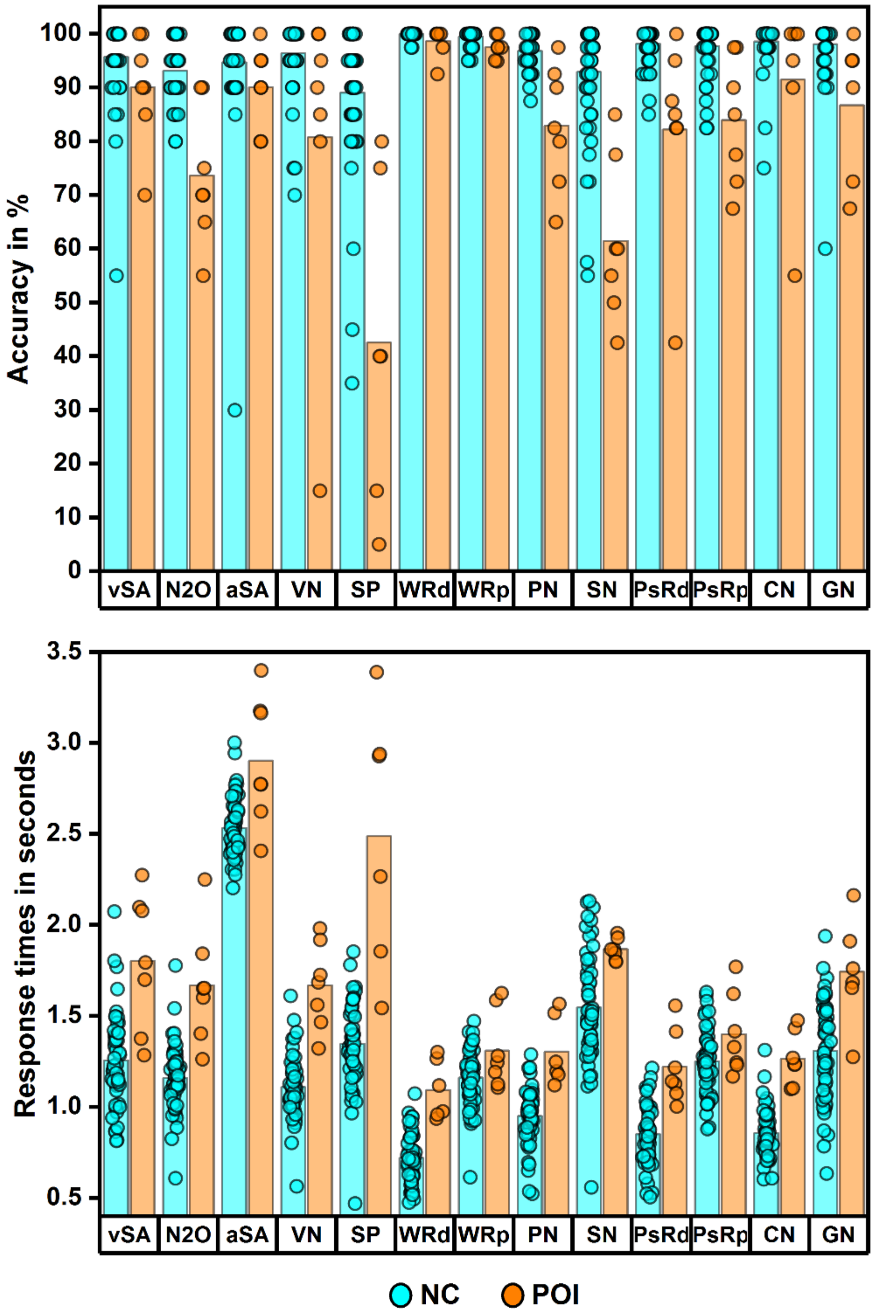

Supplementary Figure 2. In-scanner behavioural performance. Coloured circles indicate the accuracy score (top) or response time (bottom) for each neurologically-intact control (NC) and patient of interest with LpOp damage (POI). Bars highlight the respective group mean. There were seven missing response time data points distributed across the following fMRI tasks: vSA (1 NC), VN (1 NC), SP (1 NC), WRd (1 NC and P5), PN (P6) and GN (1 NC). For abbreviations of task names, see Supplementary Table 1.

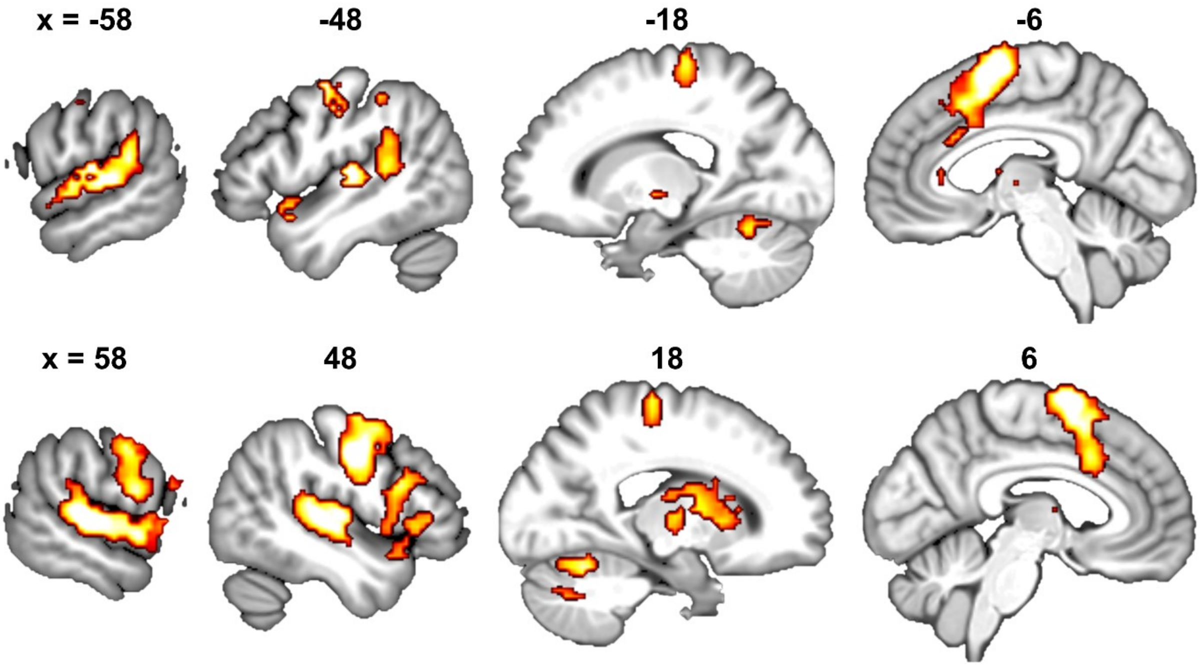

Supplementary Figure 3. Brain regions activated by both neurologically-intact controls and patients with LpOp damage during speech production. The figure depicts the network of regions recruited during accurate pseudoword reading and repetition that is common to both neurologically-intact controls and patients with LpOp damage. Areas that were activated at a voxel-level threshold of p < 0.05 FWE-corrected in controls but uncorrected in patients are shown for the purpose of data visualization only. For statistical details, see Analysis 1 in Table 2.

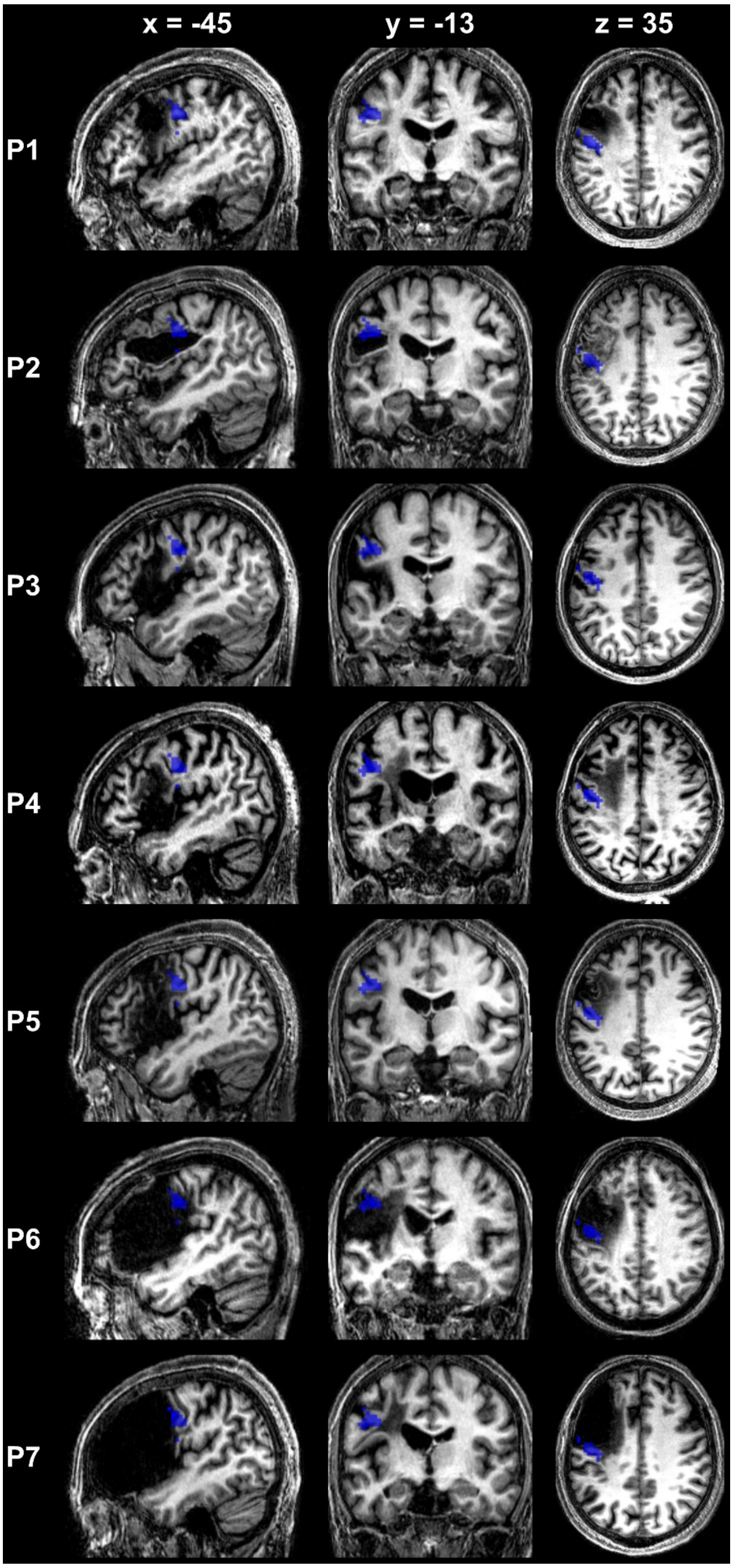

Supplementary Figure 4. Reduced activation in left sensorimotor cortex after LpOp damage. The location of the left sensorimotor region (coloured blue), where LpOp damage resulted in reduced activation during pseudoword production, is shown relative to each patient's lesion. The scans are sorted from top to bottom by the size of the patient's lesion (smallest lesion at the top and largest lesion at the bottom).

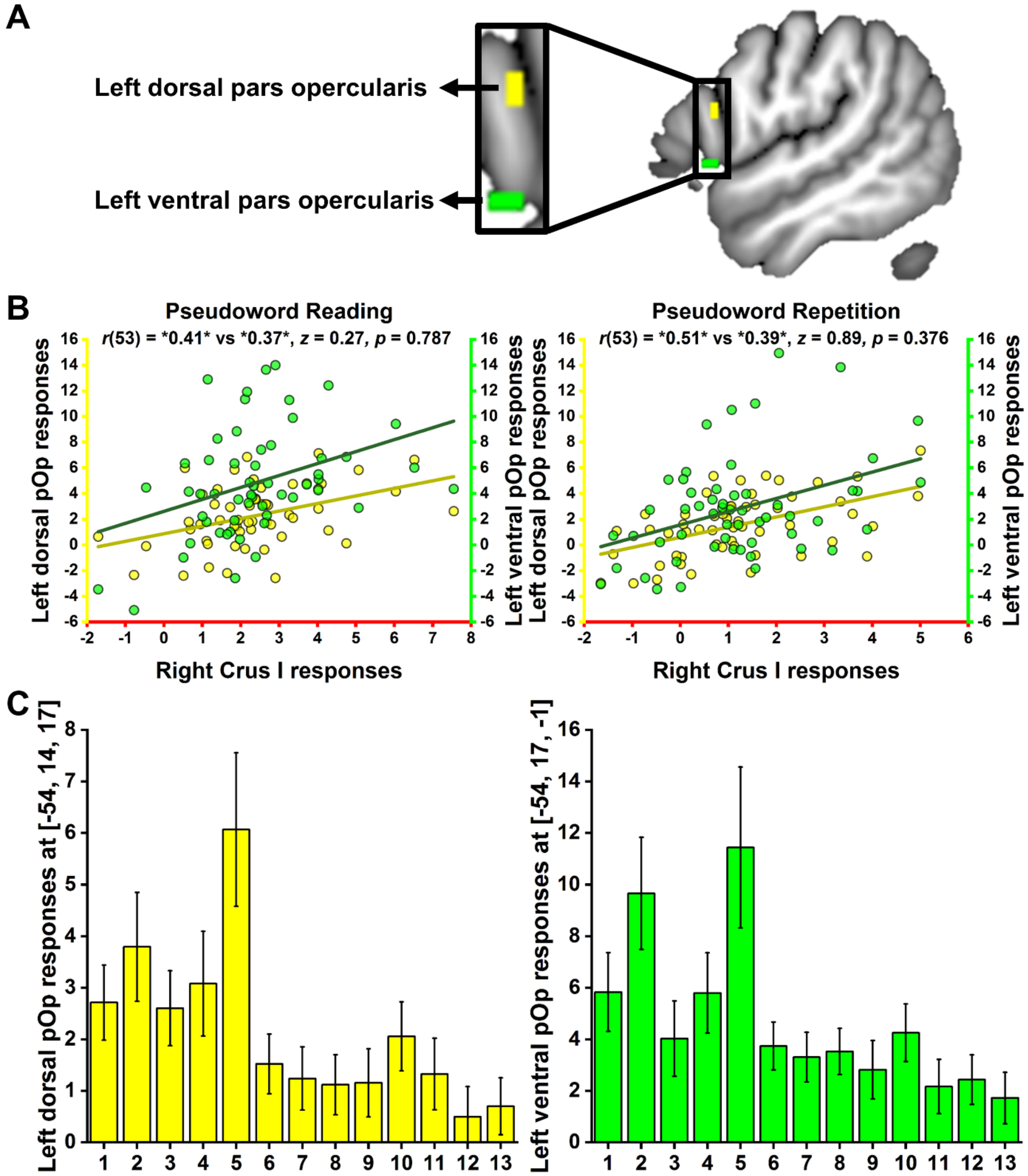

Supplementary Figure 5. Two LpOp subregions that co-activate with right cerebellar Crus I during speech production. The top panel shows the dorsal (in yellow) and ventral (in green) subregions within the left pars opercularis (pOp) where activation positively co-varied with right cerebellar Crus I activation during pseudoword reading and repetition. The middle panel shows that right cerebellar Crus I activation positively co-varied with activation in the left dorsal and ventral pOp subregions to similar degrees during pseudoword reading and repetition. Correlation coefficients surrounded by asterisks were statistically significant at $p < 0.05$. The bottom panel shows the response (mean and 95% confidence interval) profile of these two left pOp regions across 13 fMRI tasks. Numbers on the x-axis of each bar plot correspond to the following fMRI tasks: vSA, N2O, aSA, VN and SP (Experiment 1); WRd, WRp, PN, SN, PsRd, PsRp, CN and GN (Experiment 2). For abbreviations of task names, see Supplementary Table 1.
